# Prime Editing: Genome Editing for Rare Genetic Diseases Without Double-Strand Breaks or Donor DNA

**DOI:** 10.3389/fgene.2020.00528

**Published:** 2020-06-09

**Authors:** Ianis G. Matsoukas

**Affiliations:** ^1^School of Medicine, University of Bolton, Bolton, United Kingdom; ^2^René Descartes College, Athens, Greece

**Keywords:** CRISPR, genome editing, human therapeutics, prime editing, rare disease

An article published in Nature (Anzalone et al., 2019) reports the development of a genome editing experimental approach that mediates all possible base-to-base conversions, “indels,” and combinations in human genome without the need of double-strand breaks (DSBs) or donor DNA (dDNA) templates. Prime editing, the novel method of genome editing, exploits a longer-than-usual single guide RNA (gRNA), known as prime editing gRNA (pegRNA), and a fusion protein consisting of Cas9 H840A nickase fused to an engineered reverse transcriptase (RT) enzyme. Described as “search-and-replace” base-editing technology, prime editing supplies the desired genetic construct in an extension to the gRNA, which is then converted to DNA using the RT enzyme. The new approach eliminates the need for co-delivery of a corrective DNA template, performs all possible nucleotide substitutions (including those for a sizeable proportion of genetic disorders), resolves frameshifts induced by indels and confers fewer off-target edits when compared with conventional CRISPR-Cas devices. Prime editing is an exciting new complement to existing CRISPR editing systems and may even be an improvement in many cases. However, prime editing introduces new challenges. Overcoming these obstacles and applying prime editing *in vivo*, will give rise to new genome editing therapies for rare genetic diseases.

## Introduction: Programmable Genome Editing Technologies

**Figure 1:**
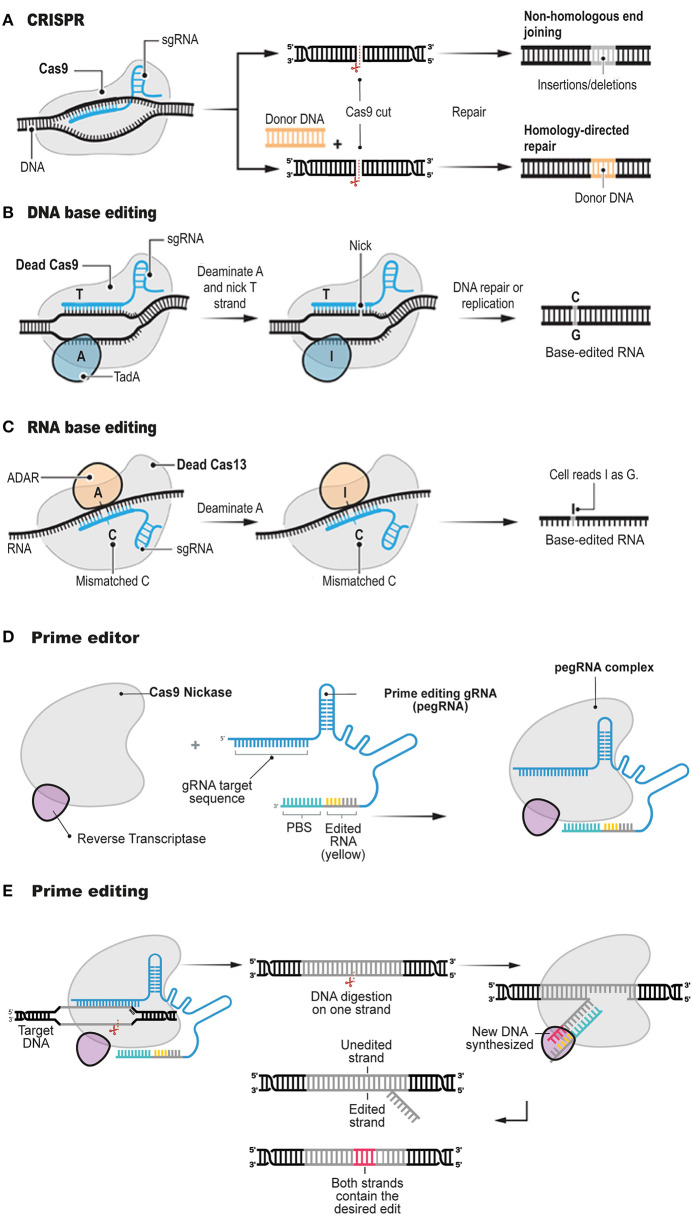
Structure and function of prime editor. The conventional CRISPR DNA editor **(A)**. CRISPR relies on the ability of CRISPR gRNAs to target the Cas9 endonuclease to precise genomic locations, where Cas9 introduces DSBs. Base editors do not digest the double strand, but instead they chemically alter single bases with deaminase enzymes such as TadA (**B**, DNA base editor) and ADAR (**C**, RNA base editor). Prime editor **(D)** involves a longer-than-usual gRNA, known as pegRNA, and a fusion protein consisting of Cas9 H840A nickase fused to a modified RT enzyme. The Cas9 element of the prime editor digest the genomic DNA and the RT element polymerises DNA onto the nicked strand based on the pegRNA sequence **(E)**. Adapted from Anzalone et al. (2019) and Matsoukas (2018a).

Precision genome editing is a versatile and powerful gene therapy tool. Since the development of CRISPR/Cas systems for genome editing (Figure 1A), the field has been subject to continuous improvements (Charpentier and Doudna, 2013; Doudna and Charpentier, 2014; Hsu et al., 2014; Sternberg and Doudna, 2015; Komor et al., 2017). The new biotechnology involves the formation of a site-specific DSB followed by two major types of repair mechanisms: non-homologous end-joining (NHEJ; Davis and Chen, 2013) and homology-directed repair (HDR; Li and Heyer, 2008). The activated type of the molecular repair mechanism depends on the genome, cellular heterogeneity and cell-division cycle (Hsu et al., 2014; Komor et al., 2017).

Therefore, the induction of a precise mutation conferred by a genome editing tool, is heavily depends on HDR occurring at the DSB *locus* via the dDNA template harboring the desired construct (Yang et al., 2014; Song and Stieger, 2017; Bollen et al., 2018). Although CRISPR/Cas systems can efficiently confer a DSB at a specific genomic sequence (Cong et al., 2013; Jinek et al., 2013; Savic et al., 2018), HDR in mammalian cells is inefficient or unsuitable due to the low innate rate of HDR and obstacles in onsite delivery of dDNA (Cong et al., 2013; Mali et al., 2013). The correction rates of the conventional genome-editing tools are 0.1–5%, and, typically, they introduce a plenty of random indels at the target genomic sequence resulting from the cellular response to DSBs (Cox et al., 2015; Hilton and Gersbach, 2015). Recently, CRISPR/Cas-mediated base editing tools have been developed to circumvent these constraints (Figures 1B,C; Komor et al., 2016; Nishida et al., 2016; Kim et al., 2017; Gehrke et al., 2018; Li et al., 2018; Sharon et al., 2018; Wang et al., 2018). Several excellent review articles have been published on the different types of customizable base editors (Rees and Liu, 2018; Molla and Yang, 2019; Yang et al., 2019). Hence, these base-editing tools will not be described in great detail here.

Briefly, base editors generate mutations at single-base resolution. All four transition mutations, C → T, G → A, A → G, and T → C, can be introduced in the genome with the already described CRISPR/Cas base editors. The cytosine base editors (Komor et al., 2016; Nishida et al., 2016; Kim et al., 2017; Gehrke et al., 2018; Li et al., 2018; Matsoukas, 2018a; Wang et al., 2018) can establish a C-G to T-A mutation, while the adenine base editors (Gaudelli et al., 2017; Kim et al., 2019) can modify an A-T base dyad into a G-C dyad. In RNA, conversion of Adenine to Inosine is also possible with an RNA base editor (Figure 1C; Cox et al., 2017; Matsoukas, 2018b). Since many genetic diseases and disorders arise from nucleotide substitutions and nucleotide additions or deletions, base editing has important implications in the study of human pathogenesis.

However, base editors cannot install all transversion mutations. For example the currently reported base editors cannot introduce the eight transversion mutations (C → A, C → G, G → C, G → T, A → C, A → T, T → A, and T → G), such as the T·A-to-A·T mutation required to precisely correct the most common etiology of sickle cell disease (SCD). SCD, a homozygous mutation (from A → T) in the sixth codon (E6V) of the human b-globin (HBB) gene, converts a glutamate to a valine which synthesizes defective b-globin proteins and results in abnormal red blood cells (Vakulskas et al., 2018). It is postulated that HDR-mediated HBB gene correction in autologous hematopoietic stem and progenitor cells, would be a safe and effective gene therapy approach for SCD.

In addition, no DSB-free experimental approach has been reported to confer targeted deletions, such as the removal of the 4-base duplication that causes Tay-Sachs disease (HEXA 1278+TATC; McGinniss et al., 2002), or targeted insertions, such as the 3-base insertion needed to precisely correct the most common etiology of cystic fibrosis (cf. Lukacs and Verkman, 2012). In regard to CF, the deletion of the phenylalanine residue at position ΔF508, is present in one or both alleles in ~90% of CF patients. Interestingly, these challenges drive the development of novel and state-of-the-art precision genome editing technologies.

This article describes prime editing, a novel genome editing tool which has been developed to expand the scope and capabilities of the existing CRISPR/Cas-based therapies for rare genetic diseases. The article also discusses the new challenges that the new biotechnology introduces and suggests possible directions for future research.

## Prime Editors: Expanding the Genome-Editing Toolbox

Targeted transversions, insertions and deletions are problematic to induce or repair efficiently and without excess by-products in most cellular types, even though they collectively account for most known pathogenic alleles. Interestingly, a paper recently published in Nature (Anzalone et al., 2019) reports a tool developed to address the base-editing limitations described above. Prime editing, the most recent base-editing tool, employs the same mechanism as conventional CRISPR/Cas systems mediating all 12 possible base-to-base conversions, and combinations, but without conferring DSBs in the target sequence or exploiting a dDNA template. Prime editing involves a longer-than-usual gRNA, known as pegRNA, and a fusion protein consisting of Cas9 H840A nickase fused to an engineered RT enzyme (Figure 1D).

This article (Anzalone et al., 2019) follows other important publications (Gaudelli et al., 2017; Kim et al., 2017) by the same research group, which described the first base-editing molecular devices. In the latest article, Anzalone et al. (2019) tested whether gRNA could be extended to include extra nucleotides. Some of these extra nucleotides would serve as a template for synthesis of a new DNA sequence, while others would bind to the DNA strand, opposite from the expected gRNA binding site, to use that genomic *locus* as a primer for the RT initiation. With the construction of the extended pegRNA, Anzalone et al. (2019) was able not only to guide Cas9 enzyme variants to the appropriate *locus* but also to install the desired edit and prime the RT enzyme (Figure 1E).

This novel experimental approach was enhanced by the construction of three incremental devices. The first molecular device (PE1) was created by a fusion of Cas9 H840A nickase and wild-type (WT) Maloney murine leukemia virus RT enzyme. In this construct, the Cas9 H840A nickase domain of the prime editing fusion protein (PEFP) nicks only one of the polynucleotide strands for subsequent restoration, whereas the RT domain generates complementary DNA (cDNA) by copying the pegRNA (carrying the desired construct) to reinstate a segment of the nicked DNA strand. In this device, Anzalone et al. (2019) also increased the length of the pegRNA site that binds the primer section of the DNA. The application of the PE1 device led to minor but still detectable genome edits.

In the PE2 device, Anzalone et al. (2019) improved the thermostability, processivity, and DNA-RNA substrate affinity of the RT component of the PEFP by introducing five specific mutations. This pentamutant RT enzyme incorporated into PE1, creating the PE2 device. The PE2 device led to a 5.1-fold improvement in prime editing point mutation efficiency and conferred targeted insertions and deletions more methodically than PE1.

In the third molecular device (PE3), Anzalone et al. (2019) introduced a second gRNA, in addition to the pegRNA. The additional gRNA was a standard gRNA directing the Cas9 H840A nickase element of the PEFP to nick the genomic DNA at a nearby site, but on the opposite strand as the original nick. They applied this approach due to a concern that efficient editing of one strand, as observed with PE2 device, might be repressed due to a mismatch between the engineered and non-engineered DNA strands. By installing a nick on the non-engineered DNA strand, Anzalone et al. (2019) reasoned that the DNA repair machinery might replace the original *locus* with the desired segment. Interestingly, PE3 device was more efficient when the additional gRNA constructed to match the newly-edited sequence introduced by the pegRNA. The PE3 device with the new characteristic of gRNA was labeled PE3b. The group also observed improved efficiencies when they designed the pegRNA to mutate the original protospacer adjacent motif (PAM).

DNA base editors have consistently proven forceful for installing precise point mutations in the genome of a wide variety of model systems [reviewed in (Sharon et al., 2018; Molla and Yang, 2019)]. In the article published by Anzalone et al. (2019), PE2, PE3, and PE3b devices were compared with cytidine/adenine base editors and with an HDR system. Interestingly, it was shown that that PE devices function in a complementary fashion to the base editor systems, depending on the desired *locus*. Also, prime editing compared favorably, in its specific abilities, to HDR. As with all genome editing technologies, base editors have the potential to operate on DNA at off-target genomic *loci*. However, it was shown that prime editing introduces much lower off-target editing than Cas9 at known Cas9 off-target *loci*.

## Prime Editing Therapeutics

Anzalone et al. (2019) tested the ability of prime editing to install and then to correct several pathogenic mutations, including the mutations known to cause SCD and Tay-Sachs diseases. In case of SCD, they exploited the PE3 device to install the HBB E6V mutation in HEK293T cell line. This approach resulted with 44% efficiency and 4.8% indels. To correct the HBB E6V allele back to WT HBB, they treated homozygous HBB E6V HEK293T cells with PE3 and a pegRNA programmed to directly revert the HBB E6V mutation to WT HBB phenotype. All tested pegRNAs devices mediated efficient correction of HBB E6V to WT HBB (26–52% efficiency), and 2.8 ± 0.70% indels. In addition, installation of a PAM-modifying silent mutation in PE3 device, improved editing efficiency and product purity to 58% correction rate, with 1.4% indels.

In regard to Tay-Sachs disease, they created the mutant phenotype by exploiting the PE3 device to install a 4-bp insertion into *HEXA*, with 31% efficiency and 0.8% indels. To recreate the WT phenotype, application of the PE3 device resulted in ≥20% editing, whereas application of the PE3b device resulted 33% efficiency with 0.32% indels.

Anzalone et al. (2019) also succeeded in introducing a mutation that imparts resistance to prion disease in humans (Mead et al., 2009) and mice (Asante et al., 2015). The PE3 device was used to install a protective G·C-to-T·A transversion into *PRION PROTEIN* in HEK293T cell line, creating a G127V mutant allele that confers the resistance to prion disease. The most effective pegRNA, by using the PE3 device, resulted in 53% installation of G127V with 1.7% indels.

The findings were further enhanced by introducing prime editing into mouse murine primary cortical neurons, using a lentiviral delivery system. To determine if prime editing is feasible in post-mitotic, terminally differentiated primary cells, they transduced primary cortical neurons from E18.5 mice with a PE3 lentiviral delivery system, in which PE2 protein components were expressed from the neuron-specific synapsin promoter along with a green fluorescence protein biomarker (Kügler et al., 2003). It was shown that the PE3 device was more efficient (7.1%), generated fewer by-products and with lower off-target editing, compared to conventional CRISPR/Cas9 genome editing systems. This indicates that post-mitotic, terminally differentiated primary cells, can tolerate prime editing.

## Discussion

The development of prime editing is a significant addition to the genome editing toolbox. Prime editing is the most recent of the tools developed to address CRISPR/Cas limitations and calibrate the genome editing process. For academic use, the technology can be currently obtained by the Addgene repository (Kamens, 2015).

Just as prime editors, cytidine base editors and adenine base editors can install transition mutations efficiently and with few indels. Arguably, there are particular cases where conventional base editors are more desired. For instance, if the target *locus* is positioned within the canonical base editing window, base editing has higher efficiency and fewer indels than prime editing. However, the application of base editing can be limited by unwanted bystander edits from the presence of multiple cytidine or adenine bases, or by the absence of a PAM positioned ~15 ± 2 nt from the target *locus*. On the other hand, prime editing eliminates the need for co-delivery of a corrective DNA construct, a factor that can magnify standard challenges in the delivery of genome editing machinery.

However, prime editing introduces new challenges. Prime editors may not be able to confer the large DNA insertions or deletions that conventional CRISPR/Cas9 systems are capable of. Also, the fact that the desired sequence has to be encoded in an extensive RNA molecule, raises concerns regarding its stability; the longer the RNA strand gets, the more likely it is to be affected by intracellular RNA-degrading enzymes. In addition, due to presence of the RT in the molecular device, random cDNAs could be potentially incorporated in the genome. Furthermore, as the protein constructs involved are too large, this might affect the delivery of a full-length therapeutic protein by a single adeno-associated viral vector.

Therefore, more work remains before prime editors can be used to treat patients with rare genetic disorders. This includes approaches for optimizing prime editors, maximizing its efficiency in different cell types, and examining potential effects of prime editing on different cell lines. Furthermore, additional experimentation with rare disease models and mechanisms at cellular and organismal level and exploring novel delivery mechanisms in animal model systems to provide potential approaches for human therapeutic applications, are required. In conclusion, undoubtedly, prime editing is another double-edged sword on offer in the field of genome editing therapies for rare genetic diseases, offering more precise base editing ability and efficiency.

## Author Contributions

The author confirms being the sole contributor of this work and has approved it for publication.

## Conflict of Interest

The author declares that the research was conducted in the absence of any commercial or financial relationships that could be construed as a potential conflict of interest.
